# Effect of Induction Chemotherapy in Nasopharyngeal Carcinoma: An Updated Meta-Analysis

**DOI:** 10.3389/fonc.2020.591205

**Published:** 2021-01-08

**Authors:** Shan-Shan Yang, Jian-Gui Guo, Jia-Ni Liu, Zhi-Qiao Liu, En-Ni Chen, Chun-Yan Chen, Pu-Yun OuYang, Fei Han, Fang-Yun Xie

**Affiliations:** ^1^ Department of Radiation Oncology, Sun Yat-sen University Cancer Center, State Key Laboratory of Oncology in South China, Collaborative Innovation Center for Cancer Medicine, Guangdong Key Laboratory of Nasopharyngeal Carcinoma Diagnosis and Therapy, Guangzhou, China; ^2^ Department of Radiation Oncology, The First People’s Hospital of Foshan, Foshan, China; ^3^ Department of Head and Neck Oncology, The Cancer Center of the Fifth Affiliated Hospital of Sun Yat-sen University, Zhuhai, China

**Keywords:** induction chemotherapy, meta-analysis, nasopharyngeal carcinoma, radiotherapy, updated

## Abstract

**Background:**

Previous meta-analysis had evaluated the effect of induction chemotherapy in nasopharyngeal carcinoma. But two trials with opposite findings were not included and the long-term result of another trial significantly differed from the preliminary report. This updated meta-analysis was thus warranted.

**Methods:**

Literature search was conducted to identify randomized controlled trials focusing on the additional efficacy of induction chemotherapy in nasopharyngeal carcinoma. Trial-level pooled analysis of hazard ratio (HR) for progression free survival and overall survival and risk ratio (RR) for locoregional control rate and distant control rate were performed.

**Results:**

Twelve trials were eligible. The addition of induction chemotherapy significantly prolonged both progression free survival (HR=0.68, 95% confidence interval [CI] 0.60–0.76, p<0.001) and overall survival (HR=0.67, 95% CI 0.54–0.80, p<0.001), with 5-year absolute benefit of 11.31% and 8.95%, respectively. Locoregional (RR=0.80, 95% CI 0.70–0.92, p=0.002) and distant control (RR=0.70, 95% CI 0.62–0.80) rates were significantly improved as well. The incidence of grade 3–4 adverse events during the concurrent chemoradiotherapy was higher in leukopenia (p=0.028), thrombocytopenia (p<0.001), and fatigue (p=0.038) in the induction chemotherapy group.

**Conclusions:**

This meta-analysis supported that induction chemotherapy could benefit patients with nasopharyngeal carcinoma in progression free survival, overall survival, locoregional, and distant control rate.

## Introduction

Nasopharyngeal carcinoma is an epithelial carcinoma arising from the nasopharyngeal mucosal lining. It is a common head and neck carcinoma in east and southeast Asia, especially in south China (1). Resulting from the non-specific symptom in early-stage disease and the absence of regular examination of nasopharynx or effective screening, almost 70% of patients have locoregionally advanced disease at the initial diagnosis (2). Radiotherapy is known to be the primary treatment approach, but it has relatively poor disease control for locoregionally advanced nasopharyngeal carcinoma. The randomized trial was initiated in 1989 to test the added value of induction chemotherapy before radiotherapy (3), and finally improved disease free survival was observed but not overall survival. As the new induction chemotherapy regimen of docetaxel and cisplatin led to significant benefit of overall survival for patients from endemic area in a phase II trial (4), more clinicians and researchers were concerned with induction chemotherapy. But what the subsequent trials (5–8) found was not always the same. A recent meta-analysis (9) concluded that induction chemotherapy had the potential to improve both progression free survival and overall survival, but it did not include another two large scale and possibly equally-weighted trials (10, 11), in which the result of induction chemotherapy on overall survival was inconsistent. Moreover, the long-term results (12, 13) of another two previous trials (7, 8) deserved great concern, especially the distinctive finding of induction chemotherapy of cisplatin and fluorouracil in 5-year overall survival (13). Therefore, an updated meta-analysis is warranted.

## Methods

### Identification of Trials and Extraction of Data

The electronic databases including PubMed, Cochrane Library, Embase, and Web of Science were searched to identify randomized controlled trials focusing on nasopharyngeal carcinoma or nasopharyngeal cancer, induction chemotherapy or neoadjuvant chemotherapy, and radiotherapy. Trials were eligible if investigating the benefit of induction chemotherapy before radiotherapy or concurrent chemoradiotherapy alone in locoregional advanced nasopharyngeal carcinoma patients.

Information of included trials was extracted, including study design, cancer staging, number of patients, year, end points, treatment protocol, follow-up duration, failure patterns and grade 3 or 4 acute toxic effects.

### Statistical Analysis

Overall, the role of induction chemotherapy in progression free survival (time from random assignment to locoregional failure, distant metastasis, or death from any cause) and overall survival (time from random assignment to death from any cause) was investigated by meta-analysis of the results of diverse trials. Hazard ratios with 95% confidence intervals (CIs) of individual trials were pooled if reported, or these were estimated as elucidated by Tierney and colleagues (14). Survival curves of pooled progression free survival and overall survival were drawn, and absolute differences between induction chemotherapy group and control group were computed as recommended by Pignon and colleagues (15). According to the method described by Tierney and Pignon, corresponding survival probability and effective number event-free of each clinical trial were obtained from the Kaplan–Meier curve at the time point of 0/12/24/36/48/60 month. And the weight of each clinical trial was also estimated. Therefore, the total survival probability of the study group and control group at each time point could be calculated. However, the role of induction chemotherapy in locoregional control and distant control was evaluated by pooling the risk ratios (RRs) with 95% CIs using the Mantel–Haenszel method (16) instead of time-dependent locoregional relapse free survival and distant metastasis free survival, because the data of hazard ratios and 95% CIs were not available at all by direct or indirect methods in certain trials. Severe acute toxicities were also pooled if available. Notably, only the data of long-term results were included for analysis. Estimation was based on random effect model in case of heterogeneity [p value of χ^2^ test <0.1 or I^2^ statistic > 50% (17)], or else fixed effect model. Finally, Begg’s test (18) and Egger’s test (19) were conducted to find potential publication bias. If publication bias was detected, the Duval and Tweedie nonparametric “trim and fill” procedure (20) was subsequently performed to further assess the possible effect.

Data analyses were done using R version 3.5.2 and Stata version 12.0 (Statacorp LP, College Station, Texas, USA). Two-sided p<0.05 was considered significant for all analyses except heterogeneity tests.

## Result

### Trials Information

**Figure 1:**
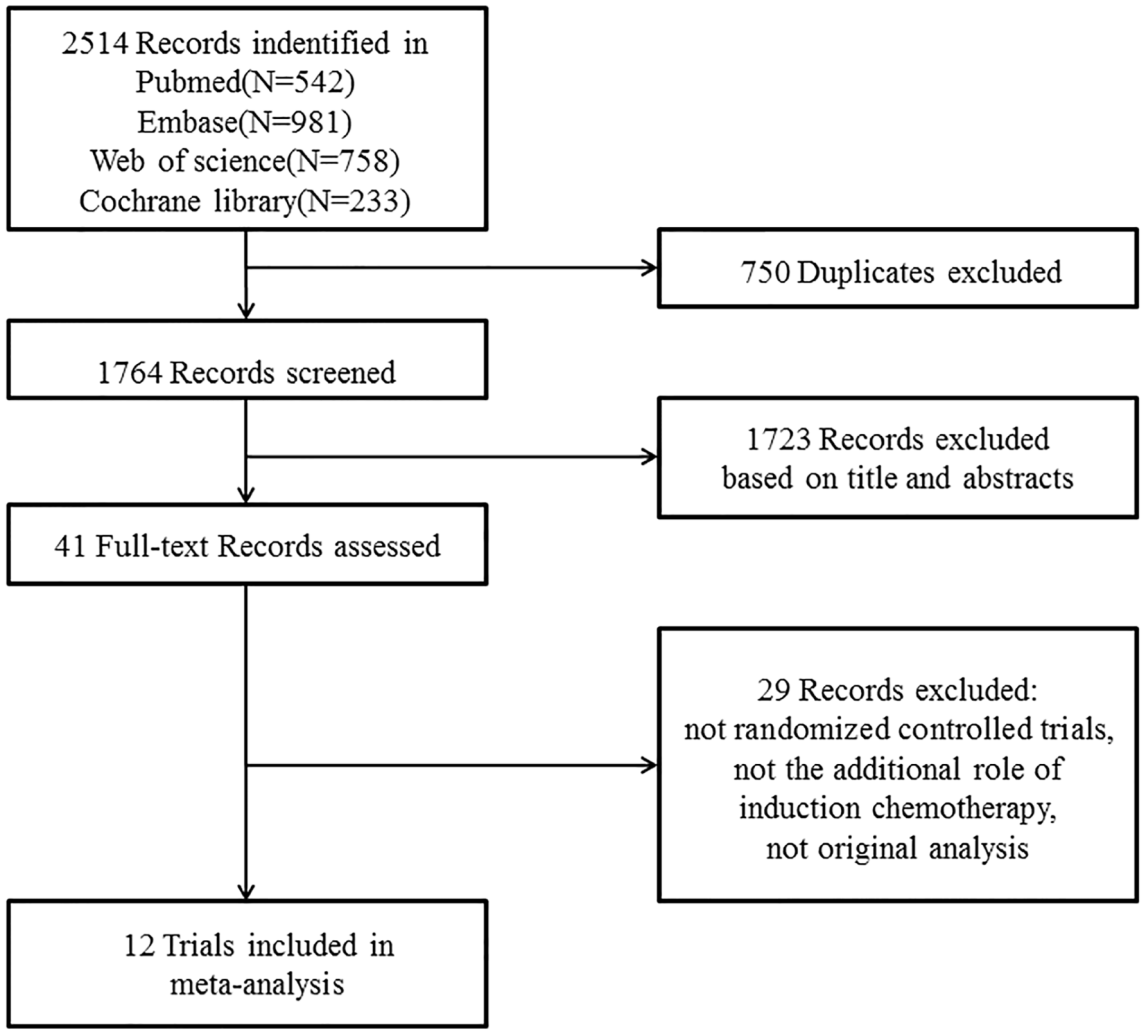
PRISMA flow diagram.

A total of 12 randomized controlled trials (3–8, 10–13, 21–24) (3,586 patients, shown in **Table 1**) were finally eligible for this meta-analysis as the literature search and trial selection (**Figure 1**) were completed. Notably, there were seven large scale trials (3, 7, 8, 10–13, 21, 22) with more than 300 participants and only three trials (4, 23, 24) enrolled fewer than 100 patients in each; 2,942 (82.04%) patients of eight trials (4, 6–8, 10–13, 21, 22) were from endemic area; two trials (7, 8) recently updated the long-term results (12, 13) and thus only one trial (21) had a median follow-up time less than three years; seven trials (4, 6–8, 10–13, 24) delivered intensity-modulated radiotherapy technique and eight trials (4–8, 10–13, 24) administrated concurrent chemotherapy of cisplatin to patients during the process of radiotherapy.

**Table 1:** Summary of included studies.

Studies	No. of patients	Stage	Inclusion period	Median follow up (m)	Radiotherapy	Induction chemotherapy	Concurrent chemotherapy
Cvitkovic et al. (3)	339	UICC any T, N2-3	1989–1993	49	2DRT:2.0Gy/f to 65–70Gy	3* BLM15mg bolus (d1)+12mg/m^2^/d(d1-5) +epirubicin70mg/m^2^(d1) +DDP 100mg/m^2^ (d1)	None
Chua et al. (21)	334	Ho’s stage III/IV and N≥3cm	1989–1993	30	2DRT:60–66Gy and additional boost for residual LNs, hypofractionated RT for most	2-3*epirubicin110mg/m^2^ +DDP60mg/m^2^	None
Ma et al. (22)	456	Chinese 1992 III–IV	1993–1994	62	2DRT:2.0Gy/f, to 68–72Gy and additional boost to 80Gy if residual	2-3* DDP100mg/m^2^(d1) +FU 800 mg/m^2^/d(d1-5) +BLM 10mg/m^2^/d(d1, d5)	None
Hareyama et al. (23)	80	All stages M0	1991–1998	49	2DRT:2.0–2.2Gy/f to 66–68Gy	2*DDP80mg/m^2^(d1) +FU800mg/m^2^(d1-4)	None
Hui et al. (4)	65	UICC1997 III–IVB	2002–2004	51.6	2DRT/3DCRT/IMRT:2Gy/f to 66Gy; residual boost of 7.5Gy, parapharyngeal boost of 20Gy	2*docetaxel75mg/m^2^ +DDP75mg/m^2^, q3w	8*DDP40 mg/m^2^ weekly
Fountzilas et al. (5)	144	UICC2002 IIB–IVB	2003–2008	55	2DRT/3DCRT:2.0Gy/f/to 66–70 Gy	3*epirubicin75mg/m^2^(d1) +paclitaxel175mg/m^2^(d1) +DDP75 mg/m^2^(d2), q3w	DDP 40 mg/m^2^ weekly
Tan et al. (6)	172	UICC1997 III–IVB	2004–2012	40.8	2DRT:70Gy/35f; IMRT:69.96Gy/33f	3*paclitaxel70mg/m^2^ +carboplatin (AUC=2.5) +gemcitabine1000mg/m^2^, q3w	DDP 40 mg/m^2^ weekly
Sun et al. (7) Li et al. (12)	480	7^th^ UICC III–IVB (except T3-4N0)	2011–2013	71.5	IMRT:2–2.35Gy/f to ≥66Gy	3*docetaxel60mg/m^2^(d1)+DDP60mg/m^2^(d1)+ 5FU600mg/m^2^(d1-5), q3w	DDP 100 mg/m^2^ q3w
Cao et al. (8) Yang et al. (13)	476	6th UICC III–IVB (except T3N0-1)	2008–2015	82.6	2DRT/IMRT: 2–2.33Gy/f to 64–72Gy	2*DDP80mg/m^2^(d1) +FU800mg/m^2^/d(d1-5), q3w	DDP 80 mg/m^2^ q3w
Frikha et al. (24)	81	UICC2002 T2b-4 and/or N1-3	2009–2015	43.1	IMRT/non-IMRT 70Gy/35f	3*Docetaxel75mg/m^2^(d1)+ DDP75mg/m^2^(d1)+ 5FU750mg/m^2^(d1-5), q3w	DDP 40 mg/m^2^ weekly
Hong et al. (10)	479	5th UICC IVa–IVB	2003–2009	72.0	1.8-2.2Gy/f, 5f/week, ≥70 Gy to the primary tumor, 66–70Gy to the involved neck	3*mitomycin8mg/m^2^, epirubicin60mg/m^2^+DDP60mg/m^2^,D1, 5-FU450mg/m^2^+ leucovorin30mg/m^2^,D8, q3w	DDP30mg/m^2^ weekly
Zhang et al. (11)	480	7th UICC III–IVb, except N0	2013–2016	42.7	IMRT 66–70Gy/30–33f	3*(gemcitabine 1g/m^2^,d1,d8+ DDP80mg/m^2^,d1),q3w	DDP100 mg/m^2^ q3w

UICC, International Union Against Cancer; BLM, bleomycin; f, fraction; LN, lymph node; civ, continuous intravenous; DDP, cisplatin; FU, fluorouracil; 2DRT, two-dimensional radiotherapy; 3DCRT, three-dimensional conformal radiotherapy; IMRT, intensity-modulated radiotherapy.

### Progression Free Survival and Overall Survival

**Figure 2:**
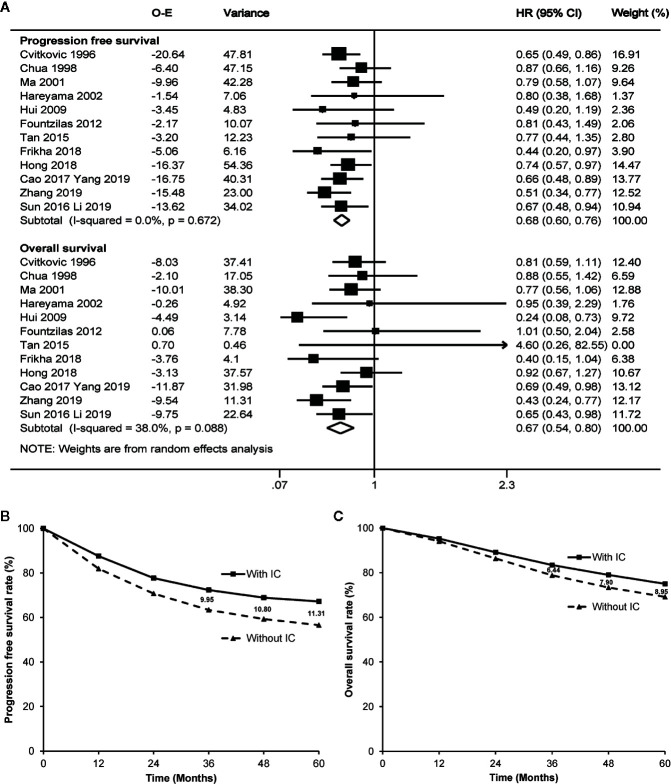
Forest plot **(A)** and estimated survival curves **(B, C)** for progression free survival and overall survival. The numbers on the figure **(B, C)** mean absolute increase of the survival rates. IC, induction chemotherapy; O-E, observed minus estimated number of events; CI, confidence interval; HR, hazard ratio.

Seven trials (4, 6, 10–13, 24) directly reported the original hazard ratio with 95% CI of progression free survival and overall survival. Based on the random effect model (potential heterogeneity for overall survival with a p value of 0.088 by χ^2^ test), meta-analysis highly favored induction chemotherapy, with a hazard ratio of 0.68 (95% CI 0.60–0.76, p<0.001) for progression free survival and a hazard ratio of 0.67 (95% CI 0.54–0.80, p<0.001) for overall survival (**Figure 2A**). The addition of induction chemotherapy before radiotherapy alone or concurrent chemoradiotherapy showed the potential to enhance the 3-year, 4-year, and 5-year progression free survival by 9.95%, 10.80%, and 11.31% (**Figure 2B**), and prolong 3-year, 4-year, and 5-year overall survival by 6.44%, 7.90%, and 8.95%, respectively (**Figure 2C**). No publication bias was observed by Egger’s test or Begg’s test (p≥0.217).

To be more careful, sensitivity analysis was conducted by excluding the four trials without concurrent chemotherapy during the phase of radiotherapy, as radiotherapy alone is no longer recommended in the clinical practice. As a result, induction chemotherapy followed by concurrent chemoradiotherapy remained the advantage of progression free survival (hazard ratio 0.64, 95% CI 0.54–0.74, p<0.001) and overall survival (hazard ratio 0.59, 95% CI 0.41–0.77, p<0.001) over concurrent chemoradiotherapy alone.

### Locoregional and Distant Control Rate

**Figure 3:**
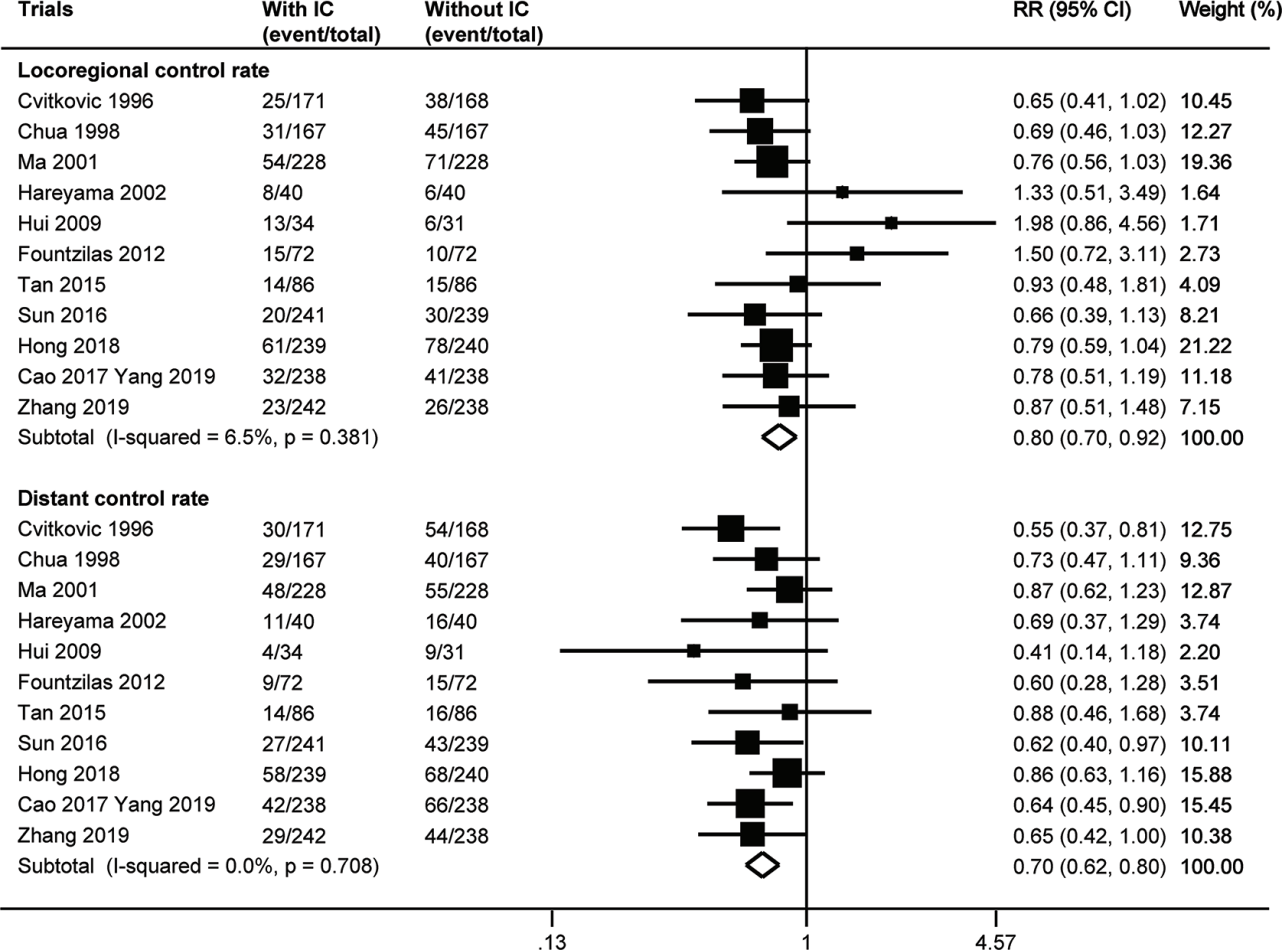
Forest plot for locoregional control rate and distant control rate. IC, induction chemotherapy; CI, confidence interval; RR, risk ratio.

The number of locoregional or distant failure was not available in the trial by Frikha et al. (24). Meta-analysis of the other 11 trials indicated that the addition of induction chemotherapy could significantly lower the risk of both locoregional and distant failure. The fixed effect model (no heterogeneity with p≥0.381 by χ^2^ test) showed a pooled risk ratio of 0.80 (95% CI 0.70–0.92, p=0.002) for locoregional control rate and a risk ratio of 0.70 (95% CI 0.62–0.80) for distant control rate (**Figure 3**). There was no evidence of publication bias for distant control rate (p≥0.185), but Egger’s test (p=0.023) and Begg’s test (p=0.024) both showed publication bias for locoregional control rate. When three theoretical missing studies were incorporated using the “trim and fill” analysis, the improved locoregional control rate resulting from the additional induction chemotherapy remained with a risk ratio of 0.75 (95% CI 0.63–0.91, p=0.003) by random effect meta-analysis (potential heterogeneity with a p value of 0.067 by χ^2^ test).

### Severe Toxicities

The main toxicities of induction chemotherapy varied by the individual regimen. Severe hematologic adverse events were not uncommon, especially neutropenia (32.74%) and leukopenia (11.94%). Unfortunately, patients were more likely to suffer from leukopenia (p=0.028), thrombocytopenia (p<0.001), and fatigue (p=0.038) during the radiotherapy phase if they had received induction chemotherapy before (**Table 2**).

**Table 2:** Grade 3 or 4 acute toxic effects.

	Cvitkovic et al. (3)	Chua et al. (21)	Ma et al. (22)	Hareyama et al. (23)	Hui et al. (4)	Fountzilas et al. (5)	Tan et al. (6)	Sun et al. (7)	Cao et al. (8)	Frikha et al. (24)	Hong et al. (10)	Zhang et al. (11)	Incidence (95% CI) or risk ratio (95% CI) with p value
**Induction chemotherapy phase (No.)**													
Total	162	155	219	40	34	63	86	239	238	40	237	239	
Anemia	NA	3	6	NA	NA	1	1	1	1	NA	16	4	1.62% (0.79%–3.28%)
Leukopenia	NA	4	8	NA	NA	NA	16	65	12	NA	139	26	11.94% (4.80%–26.73%)
Thrombocytopenia	NA	0	3	3	NA	NA	0	0	0	NA	66	13	NA
Nausea/vomiting	49.0	42	28	11	3	4	0	18	10	NA	42	48	12.88% (7.33%–19.65%)
Neutropenia	NA	NA	NA	3	33	6	50	85	35	11	NA	49	32.74% (17.74%–49.82%)
Febrile neutropenia	6.0	10	NA	NA	4	NA	NA	4	NA	3	10	NA	4.10% (2.08%–6.13%)
Fatigue	NA	NA	NA	NA	2	1	0	NA	NA	4	NA	NA	2.07% (0–5.06%)
Hair loss	NA	41	NA	NA	NA	40	0	NA	NA	6	NA	NA	20.98% (1.29%–53.74%)
renal toxicity	9.0	NA	NA	0	NA	NA	NA	0	0	NA	0	3	NA
Hepatoxicity	NA	NA	NA	NA	NA	NA	2	6	2	NA	3	5	1.45% (0.73%–2.18%)
Diarrhea	NA	NA	NA	NA	NA	NA	NA	19	1	NA	NA	1	NA
Toxic death	NA	2	0	0	NA	NA	NA	1	0	0	0	0	NA
**Radiotherapy phase (No.)**													
Total	147vs161	155vs152	219vs221	39vs40	34vs26	63vs70	86vs86	239vs238	238vs238	40vs 1	237vs227	239vs237	
Anemia	NA	NA	NA	NA	3vs5	3vs0	2vs2	4vs5	23vs9	NA	23vs2	19vs2	2.38 (0.92–6.11), p=0.072
Leukopenia	NA	NA	NA	NA	NA	16vs21	45vs32	98vs41	45vs34	NA	70vs32	47vs48	1.44 (1.04–1.98), p=0.028
Thrombocytopenia	NA	NA	NA	NA	3vs1	10vs1	12vs0	6vs2	4vs2	NA	78vs2	17vs3	6.77 (2.57–17.79), p<0.001
Neutropenia	NA	NA	NA	NA	9vs4	4vs8	21vs10	101vs17	24vs20	NA	NA	28vs25	1.67 (0.83–3.37), p=0.154
Febrile neutropenia	NA	NA	NA	NA	1vs1	0vs1	NA	7vs0	NA	NA	3vs2	1vs0	2.38 (0.89–6.35), p=0.085
Fatigue	NA	NA	NA	NA	5vs2	0vs2	12vs2	NA	NA	NA	NA	NA	2.54 (1.05–6.13), p=0.038
Nausea/vomiting	NA	NA	NA	NA	3vs2	13vs13	2vs3	106vs85	25vs21	NA	17vs30	85vs66	1.13 (0.97–1.32), p=0.105
Hepatoxicity	NA	NA	NA	NA	NA	NA	1vs0	7vs2	1vs3	NA	6vs3	1vs0	1.86 (0.83–4.15), p=0.131
Mucositis	27vs33	NA	NA	NA	8vs2	33vs38	1vs0	98vs84	16vs12	NA	82vs102	67vs76	0.96 (0.85–1.08), p=0.482
Toxic death	14vs2	NA	0vs1	0vs1	NA	NA	NA	0vs0	0vs0	NA	0vs2	0vs0	0.88 (0.10–7.36), p=0.903

NA, not available; CI, confidence interval.

## Discussion

This updated meta-analysis further confirmed the benefit of induction chemotherapy in improving progression free survival, overall survival, locoregional control, and distant control of nasopharyngeal carcinoma, based on all the currently published randomized controlled trials. Obviously, 66.28% (2,377 patients) of patients in this meta-analysis were delivered with concurrent chemoradiotherapy, which has been the standard therapy for locoregionally advanced nasopharyngeal carcinoma now; 46.26% (1,659 patients) of patients received intensity-modulated radiotherapy, which has been the primary radiation technique; 82.04% (2,942 patients) of patients were from an endemic area, which increased the applicability and practicability of the result in the real world; the median follow-up time of 52.73% (1,891 patients) of patients in four trials (10, 12, 13, 22) were longer than 60 months, which meant that the result of according meta-analysis was more likely to be robust.

The estimated overall absolute benefit of induction chemotherapy in progression free survival and overall survival provided reference basis for sample size estimation in the future randomized controlled trials. The absolute 5-year survival benefit of 11.31% in progression free survival and 8.95% in overall survival were higher than those of concurrent chemotherapy (6.6% in progression free survival and 5.3% in overall survival) or adjuvant chemotherapy (6.1% in progression free survival and 3.3% in overall survival) alone, but inferior to concurrent chemotherapy plus adjuvant chemotherapy (12.4% in progression free survival and 12.4% in overall survival), as reported in the previous meta-analysis (25). Since the high benefit of concurrent chemotherapy plus adjuvant chemotherapy was the result of taking radiotherapy alone as the control (25), induction chemotherapy thus seemed to be the most effective treatment approach. Local control and distant failure rates are, as overall survival and progression free survival, dependent on follow-up. Even if the trials are randomized with comparable follow-up in both arms, the analyses are less worthwhile than those for progression free survival and overall survival.

Overall, these trials administrated cisplatin-based induction chemotherapy regimen, with the combination of epirubicin or taxanes. In comparison with the counterparts, the former combination regimen brought about no benefit in overall survival, even in progression free survival or distant control rate for a total of more than 600 participants in the four large-scale trials (3, 5, 10, 21). Conversely, the combination of taxanes and cisplatin with or without other drugs did seem to be more effective (4, 7, 12), even though negative results were also observed in another small trial by Frikha and colleagues (24). Since this trial (24) was closed prematurely due to poor accrual, less than one third of estimated patients were actually enrolled. Perhaps it was the lack of statistic power, instead of the induction chemotherapy regimen itself, that caused the false negative finding of similar survival rate between the induction chemotherapy plus concurrent chemoradiotherapy arm and concurrent chemoradiotherapy arm in the trial (24). The newest induction chemotherapy of gemcitabine and cisplatin (11) showed highly promising results, with absolute survival benefit of 8.8% in 3-year failure free survival and 4.3% in overall survival, which appeared to be even more effective than the regimen of docetaxel, cisplatin, and fluorouracil (7). The effect of the combination of gemcitabine and cisplatin was also observed in recurrent or metastatic nasopharyngeal carcinoma (26). Certainly, another trial (6) with gemcitabine-based induction chemotherapy did not find survival improvement. This may be correlated with the combined drug of carboplatin instead of cisplatin and possible low statistic power caused by insufficient sample size resulting from overestimation of the effect of induction chemotherapy. Apart from the newly induction chemotherapy regimens, more precise eligibility criteria may be another reason for the positive efficiency of induction chemotherapy in the recent trials. As the sixth or seventh edition of AJCC/UICC staging system more effectively stratifies patients than the prior editions (27–29) and the application of magnetic resonance imaging together with fluorodeoxyglucose positron emission tomography/computed tomography more precisely staged the disease than computed tomography (30, 31), the recent trials, no wonder, more possibly enrolled high-risk patients and accordingly observed survival benefit of induction chemotherapy.

As seen in **Table 2**, induction chemotherapy itself caused common severe hematologic toxicities and meanwhile lowered the tolerance of patients to the followed concurrent chemoradiotherapy. One trial (32) showed the possibility of omitting concurrent chemotherapy after induction chemotherapy, but before more strong evidence is available, it is still the most reassuring and satisfactory if the addition of induction chemotherapy would not significantly bring down the intensity of concurrent chemoradiotherapy. Given that not all the locoregionally advanced nasopharyngeal carcinoma patients could achieve benefit from induction chemotherapy (33–35), precise selection of high-risk patients (36, 37) could be another valuable way to further enhance the magnitude of absolute benefit, apart from the exploration of newly induction chemotherapy regimen.

## Conclusion

Induction chemotherapy resulted in significant benefit in progression free survival, overall survival, locoregional control, and distant control for patients with nasopharyngeal carcinoma.

## Data Availability Statement

The original contributions presented in the study are included in the article/supplementary materials. Further inquiries can be directed to the corresponding authors.

## Author Contributions

Study concept and design: F-YX, P-YO, FH. Literature retrieval: S-SY. Data acquisition: S-SY, J-GG, J-NL, Z-QL, E-NC, C-YC. Data analysis and interpretation: S-SY, P-YO, J-GG, J-NL. Manuscript preparation and editing: S-SY, P-YO. Critical revisions: all authors. All authors contributed to the article and approved the submitted version.

## Funding 

This work was supported by the Sun Yat-sen University Clinical Research 5010 Program (2015020), the National Natural Science Foundation of China (81672665), the Sci-Tech Project Foundation of Guangdong Province (2016A020215087), and the Natural Science Foundation of Guangdong Province (2019A1515010300), Medical Scientific Research Foundation of Guangdong Province, China (Granted No. A2016197).

## Conflict of Interest

The authors declare that the research was conducted in the absence of any commercial or financial relationships that could be construed as a potential conflict of interest.
